# Cannabinoid Receptor 2 Modulates Neutrophil Recruitment in a Murine Model of Endotoxemia

**DOI:** 10.1155/2017/4315412

**Published:** 2017-08-09

**Authors:** Theodore S. Kapellos, Carlota Recio, David R. Greaves, Asif J. Iqbal

**Affiliations:** Sir William Dunn School of Pathology, South Parks Rd., Oxford OX1 3RE, UK

## Abstract

The endocannabinoid system consists of endogenous lipid mediators and cannabinoid receptors (CB) 1 and 2. It has previously been demonstrated that activation of the leukocyte-expressed CB_2_ has anti-inflammatory effects *in vivo*. Here, we report its role under baseline conditions and in a model of low-dose endotoxemia by comparing CB_2_ knockout to littermate control mice. CB_2_-deficient mice displayed significantly more neutrophils and fewer monocytes in the bone marrow under steady state. In initial validation experiments, administration of 1 mg/kg LPS to male C57BL/6J mice was shown to transiently upregulate systemic proinflammatory mediators (peaked at 2 hours) and mobilise bone marrow neutrophils and monocytes into circulation. In CB_2_ knockout mice, the level of the metalloproteinase MMP-9 was significantly elevated by 2 hours and we also observed augmented recruitment of neutrophils to the spleen in addition to increased levels of *Ccl2*, *Ccl3*, *Cxcl10*, and *Il6*. Collectively, our data show that the absence of CB_2_ receptor increases the levels of innate immune cell populations in the bone marrow under steady state. Furthermore, during an acute systemic inflammatory insult, we observe a highly reproducible and site-specific increase in neutrophil recruitment and proinflammatory chemokine expression in the spleen of CB_2_ knockout mice.

## 1. Introduction

The endocannabinoid system is an endogenous pathway which comprises two G protein-coupled (GPCRs) cannabinoid receptors (CB_1_ and CB_2_) [1, 2], the endogenous membrane phospholipid-derived ligands called endocannabinoids [3], the enzymes that synthesise and degrade them [4–7], and their transporters across cell membranes [8].

Cannabinoid receptor 1 (CB_1_) is expressed in the central nervous system predominantly by neurons [9, 10] and modulates physiological processes, such as motor behaviour, learning, memory and cognition, and pain perception [11]. In contrast, cannabinoid receptor 2 (CB_2_) is mainly expressed by immune cells in the periphery [12–14] and has been reported to possess anti-inflammatory properties in several preclinical disease models [15]. Due to the lack of psychotropic side-effects, CB_2_ agonists are considered to be a promising therapeutic strategy for the treatment of chronic inflammatory diseases, such as rheumatoid arthritis, atherosclerosis, and inflammatory bowel disease [15].

Sepsis is a systemic inflammatory syndrome initiated by Gram-negative and Gram-positive bacteria and fungi which infect the lungs, abdomen, bloodstream, and renal or genitourinary tracts [16]. Sepsis patients ultimately die of multiorgan failure which is caused by extensive tissue hypoxygenation due to ongoing microvascular leakage, disseminated intravascular coagulation, compromised energy production, and metabolic alterations [17–19]. Sepsis is characterised by an early systemic inflammatory response phase featured by symptoms, such as tachycardia, fever, hyperventilation, and activation of the complement and coagulation cascades [20, 21]. However, it is now appreciated that a compensatory anti-inflammatory response phase follows, characterised by neuroendocrine-mediated immunosuppression [22, 23].

CB_2_ activation has been explored as a potential therapeutic intervention in preclinical models of sepsis. CB_2_ agonism has been shown to ameliorate the secretion of proinflammatory cytokines and chemokines by peritoneal and splenic leukocytes and reduce the recruitment of neutrophils to the lungs [24, 25]. Similarly, fewer leukocytes adhere to small vessels in rodents treated with CB_2_-selective agonists or endocannabinoid-degrading enzyme inhibitors [26–29]. However, the literature contains conflicting reports as Csoka et al. recently reported a proinflammatory role for CB_2_ in the caecal ligation and puncture (CLP) model of sepsis [30].

MMP-9 is a member of the enzyme family of metalloproteinases and catalyses the degradation of extracellular matrix proteins. It has been previously described to mediate tissue remodelling under physiological and pathophysiological conditions, and its expression is upregulated to stimulate immune responses in diseases, such as arthritis, diabetes, and cancer [31]. A MMP-9-induced immune function that has been extensively studied in the past is neutrophil transmigration across basement membrane [32]. *In vivo*, MMP-9 release by nonhematopoietic cells drives neutrophil recruitment to influenza virus-infected airways [33] and promotes neutrophil and T cell mobilisation to the postischemic liver in mice [34]. Similarly, MMP-9 deletion protects mice from endotoxic shock and sepsis; therefore, MMP-9 inhibition has been proposed as a potential therapeutic approach to treat human sepsis [35].

In the present study, we demonstrate that the myeloid compartment of the bone marrow which provides the periphery with leukocytes during systemic inflammation is dysregulated in CB_2_ knockout mice under steady state. We went on to study the effects of CB_2_ deficiency on the kinetic parameters of proinflammatory mediator production and leukocyte mobilization in a low-dose endotoxemia model. We found that the absence of CB_2_ results in increased levels of MMP-9 in the serum at 2 hours and enhanced neutrophil recruitment to the spleen. Collectively, our data suggest that this GPCR modifies immune cell migration to peripheral tissues in the context of acute systemic inflammatory response.

## 2. Materials and Methods

### 2.1. Materials

FBS, LPS from *E. coli* (O127:B8), HEPES, BSA, heparin, and paraformaldehyde (PFA) were purchased from Sigma-Aldrich (Gillingham, UK). PBS was from Lonza (Slough, UK). EDTA was purchased from VWR technologies (East Grinstead, UK). HBSS was purchased from Life Technologies (MA, USA).

### 2.2. Animals

All animal studies were conducted with ethical approval from the Dunn School of Pathology Animal Welfare Ethical Review Board and in accordance with the UK Home Office regulations (Guidance on the Operation of Animals, Scientific Procedures Act, 1986). Male 8- to 10-week-old C57BL/6 mice were purchased from the Biomedical Services Unit (Oxford, UK) and were housed in a 12-hour light/12-hour dark cycle unit with free access to food and water. CB_2_ knockout animals backcrossed five times to C57BL/6 genetic background were purchased from the Jackson Laboratory (ME, USA) and were further backcrossed for an additional five generations to C57BL/6 mice before use. Power calculations were carried out prior to all *in vivo* experiments to determine the minimum number of animals needed to detect an effect of at least 30% with *p* < 0.05 between wild-type and CB_2_ knockout mice.

### 2.3. Endotoxemia Model

Male C57BL/6J and CB_2_ knockout mice were injected intraperitoneally (i.p.) with 1 mg/kg LPS and were monitored until sacrifice at 1, 2, 4, and 8 hours. Naïve animals were used for the steady state measurements. All animals were euthanised via asphyxiation with a rising concentration of CO_2_. The peritoneal cavities were lavaged with 5 ml ice-cold PE (PBS/2 mM EDTA) buffer and blood was retrieved from the hepatic vein into heparin- (10 U/ml-) treated tubes. Blood was left to clot for 5 hours at 4°C and serum was collected after a 10 min centrifugation at 8000 ×g. The lungs, spleen, and bone marrow were harvested and stored on ice until further processing.

### 2.4. Tissue Processing

Lungs were homogenised and were incubated for 1 hour in 1 mg/ml Collagenase D (Roche, Welwyn Garden City, UK) at 37°C/5% CO_2_. The homogenates were then passed through 70 *μ*m cell strainers and were prepared for flow cytometry.

Spleens were cut into 75 mm^3^ pieces and digested enzymatically in collagenase D, while bone marrow cells were flushed from murine femora in 10 ml PBS. The lysates were resuspended in 1 ml PBS, and 200 *μ*l were mixed with 2 ml BD Pharm Lyse buffer (BD Biosciences, Oxford, UK) for 15 min at room temperature to lyse red blood cells. Cells were then washed twice with 1% BSA in PBS and were stained according to the flow cytometry protocol.

Fresh blood (50 *μ*l) were stained according to the flow cytometry protocol and red blood cells were lysed with the BD FACS lysis solution (BD Biosciences) for 5 min at room temperature. Samples were then washed twice with FACS buffer.

### 2.5. Flow Cytometry

Harvested cells were blocked with 5% FBS in PBS for 15 min on ice and were then stained with anti-CD45 (30-F11; BD Pharmigen), anti-Ly-6G (1A8; BD Pharmigen), anti-Ly-6G (1A8; Biolegend), anti-Ly-6B.2 (7/4; Abd Serotec), anti-CD11b (M1/70; Biolegend), anti-Ly-6C (HK1.4; Biolegend), and anti-CD115 (AFS98; Biolegend) at 2 *μ*g/ml in FACS buffer (PBS; 2%FBS, 25 mM HEPES, 5 mM EDTA) for 30 min on ice protected from light. Cells were pelleted at 5000 × g for 10 min and resuspended in 1% PFA. Samples were run on a Dako Cyan ADP flow cytometer (Beckman Coulter Ltd., High Wycombe, UK) and analysed with FlowJo v10.0.8 software (Tree Star Inc., Ashland, USA).

### 2.6. Cytokine, Chemokine, and Growth Factor Level Measurement

In the time course experiment, cytokine and chemokine serum levels were measured by ELISA as instructed by the manufacturer (R&D systems, Abingdon, UK). Comparison of WT and CB_2_ knockout animal serum cytokine and chemokine levels were assessed by a Magnetic Luminex Screening Assay as instructed by the manufacturer (R&D systems) at a Bio-Plex 200 system (Bio-Rad, Hemel Hempstead, UK). Granulocyte colony-stimulating factor (G-CSF) levels in the serum of WT, and CB_2_ knockout animal was quantified with a Quantikine ELISA (R&D systems) following the instructions of the manufacturer. All samples were diluted in reagent diluent to be in the linear part of the standard curve.

### 2.7. qPCR

RNA extraction was carried out with the RNeasy kit (Qiagen, Manchester, UK), and RNA quality was verified with a ND-100 spectrophotometer (Nano Drop Technologies, DE, USA). cDNA was synthesized from 400 to 600 ng RNA using the QuantiTect Reverse Transcription kit (Qiagen) according to the manufacturer's instructions. cDNA (20–30 ng) was used as a template in qPCR experiments using specific primers (500 nM) and 2X Sybr Select (Life Technologies) as the detection chemistry. The qRT-PCR thermal profile consisted of one step at 95°C for 5 min, one step of 40 cycles of 95°C for 20 s, 60°C for 20 s, and 72°C for 20 s, and the final elongation step at 72°C for 5 min. Melt curve analysis was run after every experiment. The experiments were carried out with a Step One Plus platform (Applied Biosystems, MA, USA) and analysed with the StepOne software. *Il6*, *Ccl3*, *Cxcl10*, and *Cmtm6* primer pairs were purchased from Qiagen. *Actg1* was the chosen reference gene (Table 1). Cycle threshold (Ct) values were determined, and relative mRNA contents were inferred from normalization of the gene of interest expression to that of the housekeeping gene (ΔCt). Relative expression results were plotted as 2^(−ΔCt).

### 2.8. Cell Counts

To calculate the number of leukocytes in blood and tissues, 300 *μ*l of samples were mixed 12.5 *μ*l with CountBright Absolute Counting Beads (Life Technologies) and were run on a Dako Cyan ADP flow cytometer. Numbers were determined from the cell : bead ratio on the forward/side scatter flow cytometry plot as instructed by the manufacturer.

### 2.9. PCR Arrays

The Mouse Chemokines & Receptors PCR array (Qiagen) was used as instructed by the manufacturer. Briefly, pooled RNA samples (400 ng each) from 9 wild-type and CB_2_ knockout murine spleens were reversed transcribed using the QuantiTect Reverse Transcription kit (Qiagen), and the RT^2^ qPCR mastermix (including cDNA) was aliquoted across the provided PCR arrays. A fast protocol was followed consisting of one step at 95°C for 10 min and one step of 40 cycles of 95°C for 15 s and 60°C for 1 min. Melt curve analysis was run after every experiment. Experiments were carried out with the Step One Plus platform (Applied Biosystems) and analysed with the StepOne software. Relative expression against endogenous *Gapdh* was plotted as 2^(−ΔCt).

### 2.10. Statistical Analysis

All data are reported as mean + SEM of several independent experiments. Statistical analysis was carried out with GraphPad Prism 6.0 (CA, USA). A Grubbs' test was performed before statistical analysis to remove significant outliers from the datasets (GraphPad Prism). A student *t*-test was used to analyse experiments with two sets of normally distributed data, whereas two-way ANOVA with Sidak's post hoc multiple comparisons test was used to assess the influence of two independent categorical variables in experiments with one continuous dependent variable. Results were considered significant when *p* < 0.05.

## 3. Results

### 3.1. Neutrophils and Monocytes Are Recruited to the Lungs and Peritoneal Cavity upon LPS Administration

**Figure 1:**
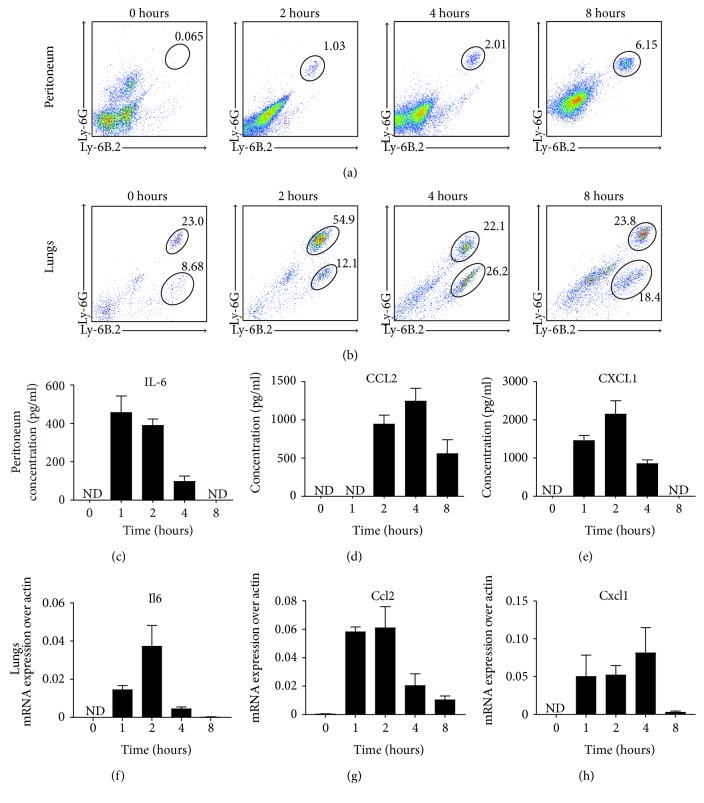
Immune cell recruitment to peripheral tissues is maximal at 2 hours post LPS challenge. Male C57BL/6J mice (8–10 weeks old) were administered i.p. with 1 mg/kg LPS and innate immune cell recruitment to peripheral tissues, and production of proinflammatory mediators was followed for 8 hours. Naïve animals were used for the steady state measurements. Peritoneal lavage fluid (a) and lungs (b) were harvested to assess the presence of neutrophils (CD45^+^Ly-6G^hi^Ly-6B.2^+^) and monocytes (CD45^+^Ly-6G^mid^Ly-6B.2^+^) by flow cytometry. Representative dot plot graphs gated on CD45^+^ cells are shown for the peritoneum (a) and lungs (b). The levels of the cytokine IL-6 (c) and chemokines CCL2 (d) and CXCL1 (e) were measured in peritoneal fluid by ELISA. The mRNA levels of *Il6* (f), *Ccl2* (g), and *Cxcl1* (h) in lung homogenates were measured by qRT-PCR. Data are from one experiment with 5-6 mice per time point. Mean + SEM are represented in all bar graphs. ND: not detected.

We first carried out a time course evaluation of innate immune cell recruitment to peripheral tissues in order to understand the cellular kinetics in the endotoxemia model. We therefore administered i.p. 1 mg/kg LPS into male C57BL/6J mice sacrificed at 1, 2, 4, and 8 hours. As shown in Figure 1(a), neutrophils (CD45^+^Ly-6G^hi^Ly-6B.2^+^) infiltrated the peritoneum at 2 hours and were found at all subsequent time points studied. Similarly, neutrophil and monocyte (CD45^+^Ly-6G^mid^Ly-6B.2^+^) populations infiltrated the lungs at the 2-hour time point (Figure 1(b)). Neutrophils were also detected in the livers of endotoxemic mice from 2 hours (data not shown).

We next sought to assess the inflammation score in these organs. We chose IL-6 because it has been shown to be a good predictor of disease progression and mortality in humans [36, 37], CCL2 as the main chemokine responsible for inflammatory monocyte recruitment to inflamed tissues [38, 39] and CXCL1, CXCL2, and CXCL5 as the murine analogues of human IL-8 which control neutrophil migration to injury sites [40]. Proinflammatory mediators in the peritoneal fluid of endotoxemic mice followed different kinetic patterns, and their levels peaked between 2 and 4 hours upon LPS administration. Subsequently, they decreased with IL-6 and CXCL1 levels falling below the detection limit (Figures 1(c), 1(d), and 1(e)). In the lungs, the mRNA levels of *Il6* and *Ccl2* peaked at 2 hours and decreased by 8 hours, whereas *Cxcl1* expression peaked at 4 hours (Figures 1(f), 1(g), and 1(h)).

Collectively, these observations show that low-dose LPS administration induces the recruitment of neutrophils and monocytes to peripheral tissues where a range of proinflammatory mediators are released. The pattern of leukocyte recruitment displays a continuous increase trend, whereas inflammatory mediator production peaks at 2 hours and is decreased until 8 hours post LPS administration.

### 3.2. Characterisation of Proinflammatory Mediator Production during Endotoxemia Time Course

**Figure 2:**
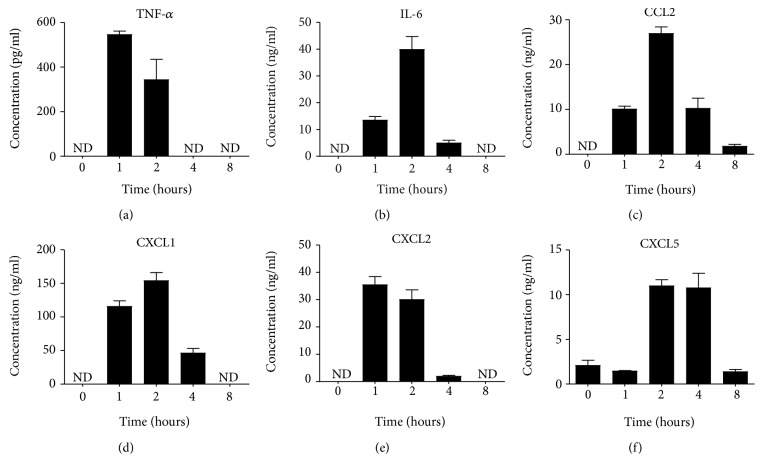
Proinflammatory mediator levels peak at 2 hours post LPS challenge. Male C57BL/6J mice (8–10 weeks old) were administered i.p. with 1 mg/kg LPS and the levels of proinflammatory mediators in the serum was measured up to 8 hours post challenge. The levels of TNF-*α* (a), IL-6 (b), CCL2 (c), CXCL1 (d), CXCL2 (e), and CXCL5 (f) were measured in the serum by ELISA. Data are from one experiment with 5-6 mice per time point. Mean + SEM are represented in all bar graphs. ND: not detected.

We next looked into the systemic levels of proinflammatory mediators at 1, 2, 4, and 8 hours post LPS. Measurement of proinflammatory cytokines and chemokines showed that all mediators apart from TNF-*α* reach a peak at 2 hours post LPS administration and are subsequently reduced as shown at the 8-hour time point (Figure 2). TNF-*α* levels peaked at 1 hour and they were detectable until the 2-hour time point (Figure 2(a)), while IL-6, CCL2, CXCL1, and CXCL2 were still present at later time points (Figures 2(b), 2(c), 2(d), and 2(e)). Finally, one of the three analogues of human IL-8 in mice, CXCL5, displayed a transient secretion pattern in the endotoxemic serum between 2 and 4 hours (Figure 2(f)), while the anti-inflammatory cytokine IL-10 was undetectable at all selected time points (data not shown). Taken together, our data suggest that proinflammatory mediators in the circulation are rapidly upregulated within the first 2 hours upon LPS administration.

### 3.3. CB_2_ Deficiency Does Not Regulate Cytokine or Chemokine Secretion but Significantly Augments MMP-9 Levels

To test whether a functional CB_2_ receptor has the ability to modulate proinflammatory mediator secretion in the endotoxemic serum, we injected 1 mg/kg LPS into male C57BL/6J and CB_2_ knockout mice for 2 hours. We hypothesised that if CB_2_ ameliorated disease severity, CB_2_-deficient mice would have elevated proinflammatory mediator levels in their serum at the 2-hour time point.

**Figure 3:**
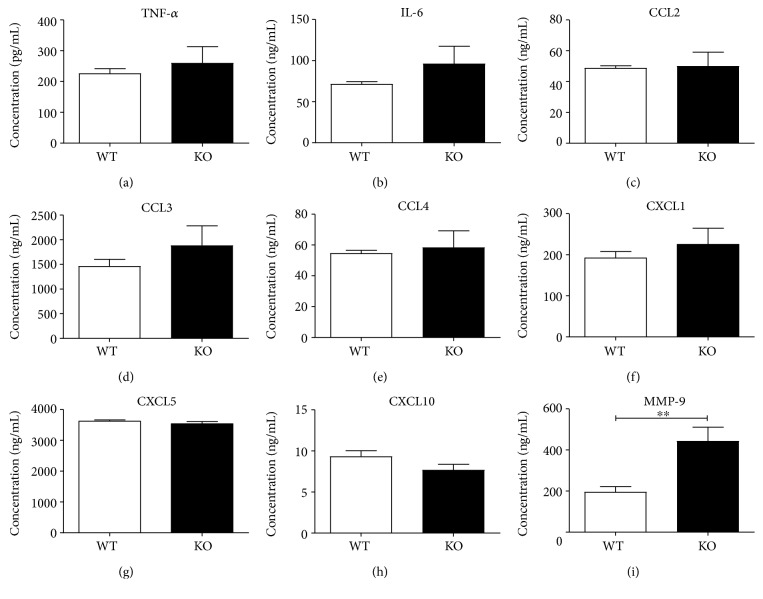
CB_2_ deficiency results in higher MMP-9 levels in the serum of endotoxemic mice. Male C57BL/6J and CB_2_ knockout mice (8–10 weeks old) were administered i.p. with 1 mg/kg LPS and the levels of proinflammatory mediators in the serum at 2 hours was measured. The levels of TNF-*α* (a), IL-6 (b), CCL2 (c), CCL3 (d), CCL4 (e), CXCL1 (f), CXCL5 (g), CXCL10 (h), and MMP-9 (i) were measured in serum samples by Luminex. Data are from two independent experiments with 6–9 mice per group and 3–5 mice per group per experiment. Mean + SEM are represented in all bar graphs and data were analysed with a one-tailed student *t*-test, ^∗∗^*p* < 0.01.

The concentrations of cytokine and chemokine mediators evaluated in the serum of C57BL/6J and CB_2_ knockout mice were comparable (Figures 3(a), 3(b), 3(c), 3(d), 3(e), 3(f), 3(g), and 3(h)). Interestingly, secreted metalloproteinase MMP-9, which has a role in neutrophil migration [32], was significantly upregulated in the serum of CB_2_ knockout animals (*p* < 0.01). This finding suggested that CB_2_ may be regulating neutrophil recruitment in this acute model of inflammation. We therefore decided to examine neutrophil infiltration to peripheral tissues.

### 3.4. CB_2_ Genetic Ablation Does Not Affect Neutrophil Recruitment to the Lungs

**Figure 4:**
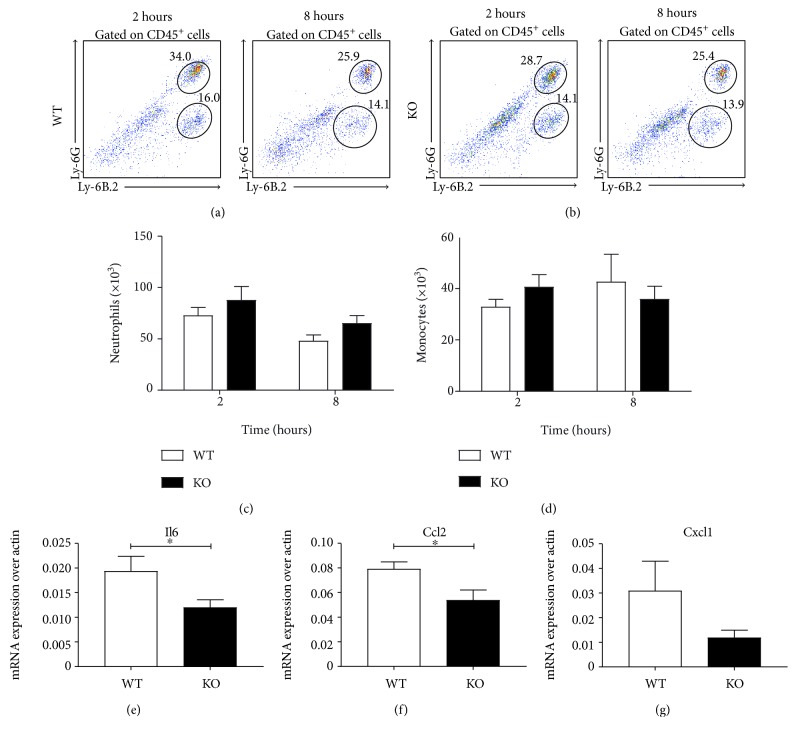
CB_2_ knockout mice have comparable numbers of neutrophils and monocytes in the lungs following LPS challenge. Male C57BL/6J and CB_2_ knockout mice (8–10 weeks old) were administered i.p. with 1 mg/kg LPS and innate immune cell recruitment to the lungs was studied at 2 and 8 hours. Lung homogenates were stained for neutrophils (CD45^+^Ly-6G^hi^Ly-6B.2^+^) and monocytes (CD45^+^Ly-6G^mid^Ly-6B.2^+^) by flow cytometry. Representative dot plot graphs gated on CD45^+^ cells are shown from one C57BL/6J (a) and one CB_2_ knockout (b) mouse for each time point. Pooled data from two independent experiments with 8-9 mice per group are shown for neutrophils (c) and for monocytes (d). (e–g) Proinflammatory mediator mRNA expression was measured in lung homogenates from wild-type and CB_2_ knockout murine lungs at 2 hours by qRT-PCR. Data for *Il6* (e), *Ccl2* (f), and *Cxcl1* (g) are pooled from two independent experiments with 8-9 mice per group and 4-5 mice per group per experiment. Mean + SEM are represented in all bar graphs and data were analysed with a one-tailed student *t*-test (e–g) or a two-way ANOVA with Sidak's post hoc multiple comparisons test (b, d), ^∗^*p* < 0.05.

The lungs are routinely selected as the major site of leukocyte recruitment in sepsis models. Assessment of neutrophil and monocyte recruitment to the lungs at 2 hours revealed comparable numbers of neutrophils (CD45^+^Ly-6G^hi^Ly-6B.2^+^) and monocytes (CD45^+^Ly-6G^mid^Ly-6B.2^+^) in both wild-type and CB_2_ knockout mice (Figures 4(c) and 4(d)). Similar observations were made at the later time point of 8 hours (Figures 4(c) and 4(d)). We analysed the inflammation score in this tissue by measuring the mRNA levels of proinflammatory mediators in lung homogenates at both time points. We found that *Il6* (Figure 4(e)) and *Ccl2* (Figure 4(f)) levels are significantly downregulated in the CB_2_ knockout lungs (*p* < 0.05), while *Cxcl1* displayed a nonsignificant reduction trend (Figure 4(g)). These data suggest that CB_2_ does not regulate neutrophil infiltration to the lungs during acute systemic inflammation.

### 3.5. CB_2_ Knockout Animals Have a Bigger Neutrophil Population in the Bone Marrow under Steady State

The bone marrow plays an integral part in sepsis by replenishing leukocyte numbers in the circulation via G-CSF-triggered emergency myelopoiesis [41]. To investigate the role of this tissue in neutrophil and monocyte mobilisation in the absence of CB_2_, we harvested bone marrow from male C57BL/6J and CB_2_ knockout mice femora following endotoxemia for 2 and 8 hours and counted the numbers of the immune cell populations by flow cytometry. Naïve mice served as the steady state control.

**Figure 5:**
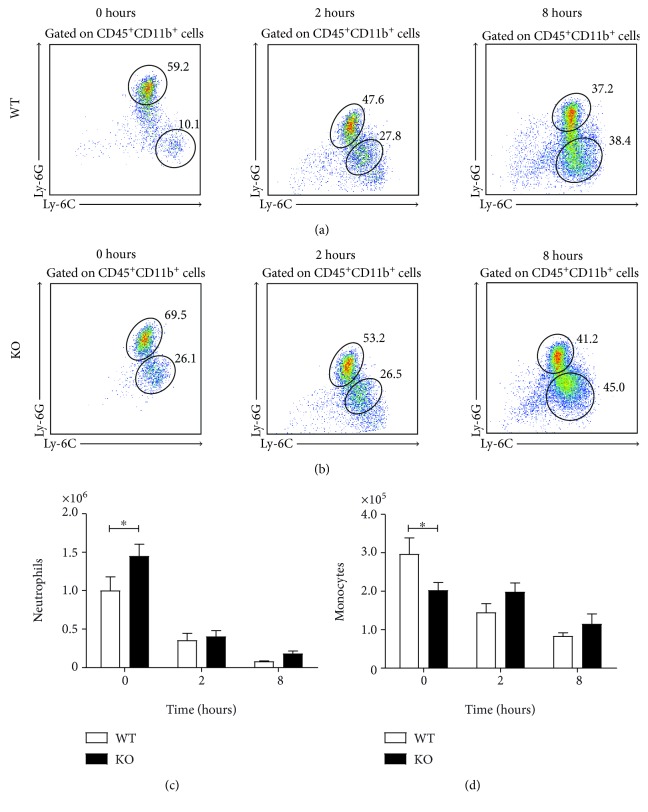
CB_2_ knockout mice display elevated neutrophils and monocytes in the bone marrow compared to littermate controls. Male C57BL/6J and CB_2_ knockout mice (8–10 weeks old) were administered i.p. with 1 mg/kg LPS and innate immune cell population numbers in the bone marrow were assessed for up to 8 hours. Flushed bone marrow cells from the animal femora were stained for neutrophils (CD45^+^CD11b^+^Ly-6G^hi^Ly-6C^+^) and monocytes (CD45^+^CD11b^+^Ly-6G^mid^Ly-6C^+^) by flow cytometry. Representative dot plot graphs from one C57BL/6J (a) and CB_2_ knockout (b) mouse gated on CD45^+^CD11b^+^ cells are shown for the full time course. Pooled data from two independent experiments with 7–10 mice per group and 3–5 mice per group per experiment are shown for neutrophils (c) and monocytes (d). Mean + SEM are represented in all bar graphs and data were analysed with a two-way ANOVA with Sidak's post hoc multiple comparisons test, ^∗^*p* < 0.05.

Bone marrow assessment under steady state revealed differences in neutrophil (CD45^+^CD11b^+^Ly-6G^hi^Ly-6C^+^) and monocyte (CD45^+^CD11b^+^Ly-6G^mid^Ly-6C^+^) populations (Figures 5(a) and 5(b)). CB_2_-deficient mice had significantly more neutrophils (*p* < 0.05) and significantly fewer monocytes (*p* < 0.05) in the bone marrow in comparison with their control littermates (Figures 5(c) and 5(d)). During endotoxemia, the numbers of neutrophil and monocyte in bone marrow were sharply reduced (*p* < 0.0001) in both C57BL/6J and CB_2_ knockout mice (Figures 5(c) and 5(d)). In particular, neutrophil numbers declined to 35% and 8% in wild-type mice at 2 and 8 hours, respectively, upon LPS administration, whereas their numbers fell to 28% and 13% in CB_2_ knockout mice (Figure 5(c)). Monocytes decreased by 28% at 8 hours in wild-type mice, in stark contrast to CB_2_ knockout mice where a reduction of 57% was observed (Figure 5(d)).

### 3.6. Increased Neutrophil Mobilisation in CB_2_ Knockout Mice Spleens

Apart from the bone marrow, neutrophils have also been reported to reside in other tissues, such as the spleen and the liver where marginated populations are in a two-way equilibrium with the bloodstream [42, 43]. The substantial egress of neutrophils from the bone marrow at the peak 2 h time point suggested that there might be other peripheral organs where neutrophil recruitment might be dysregulated in CB_2_ knockout mice during endotoxemia. For this reason, we injected 1 mg/kg LPS to male C57BL/6J and CB_2_ knockout mice for 2 and 8 hours and counted neutrophil numbers in the blood, peritoneal cavity, and spleen by flow cytometry.

Neutrophil (CD45^+^CD11b^+^Ly-6G^hi^Ly-6C^+^) numbers in the blood were comparable between the two genotypes under steady state and they were significantly (*p* < 0.0001) elevated during endotoxemia. Nevertheless, we did not observe a statistically significant difference between C57BL/6J and CB_2_ knockout mice at any time point studied. Congruent with this, serum G-CSF levels were comparable between the two genotypes (data not shown). We next measured neutrophil (CD45^+^Ly-6G^hi^Ly-6B.2^+^) levels in the peritoneal cavity as the site of sterile infection; however, numbers were also similar between wild-type and CB_2_ knockout mice (data not shown).

**Figure 6:**
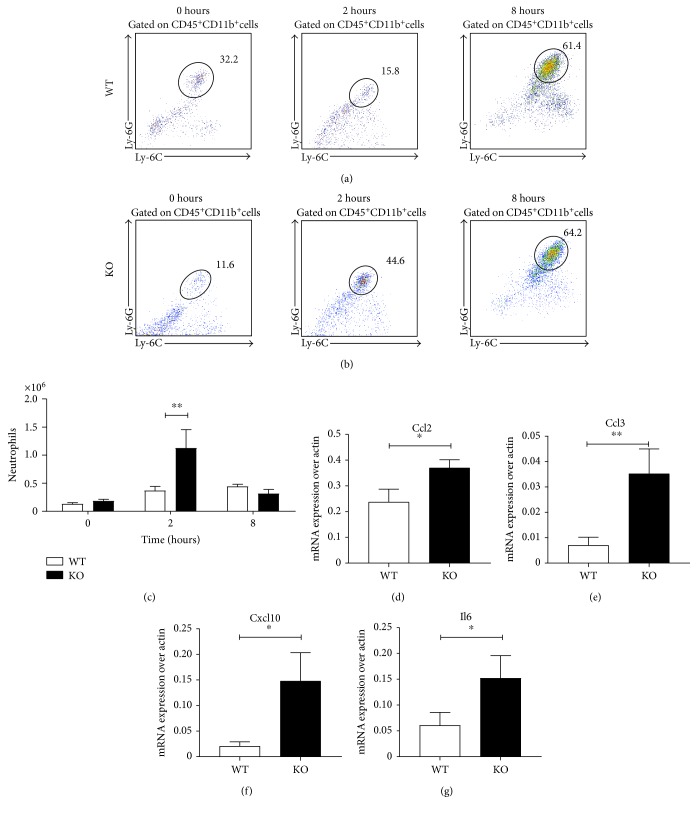
CB_2_ knockout mice have increased neutrophils in the spleen at 2 hours following LPS challenge. Male C57BL/6J and CB_2_ knockout mice (8–10 weeks old) were administered i.p. with 1 mg/kg LPS for 8 hours and neutrophil numbers in the spleen were assessed for 8 hours. Spleen homogenates were stained for neutrophils (CD45^+^CD11b^+^Ly-6G^hi^Ly-6C^+^) by flow cytometry. Representative dot plot graphs from one C57BL/6J (a) and one CB_2_ knockout (b) mouse gated on CD45^+^CD11b^+^ cells are shown for the full time course. Pooled data from two independent experiments with 7–10 mice per group are shown for neutrophils in (c). Mean + SEM are represented in the bar graph, and data were analysed with two-way ANOVA with Sidak's post hoc multiple comparisons test, ^∗∗^*p* < 0.01. The mRNA levels of (d) *Ccl2*, (e) *Ccl3*, (f) *Cxcl10*, and (g) *Il6* were tested by qPCR. Data are pooled from two independent experiments with 8-9 mice per group and 4-5 mice per group per experiment. Mean + SEM are represented in all bar graphs and data were analysed with a one-tailed student *t*-test, ^∗^*p* < 0.05, ^∗∗^*p* < 0.01.

We finally looked at the population of splenic neutrophils between wild-type and CB_2_ knockout mice during endotoxemia (Figures 6(a), 6(b), and 6(c)). Acute splenitis has been documented in necropsies from human septic patients [44], and this tissue has been reported to play a crucial role in clearance of pathogens and activation of adaptive immunity in sepsis [45, 46]. At 2 hours, we observed a significant (*p* < 0.05) increase in neutrophil levels in the spleens of CB_2_ knockout mice. Interestingly, at 8 hours, the splenic neutrophil numbers were comparable between wild-type and CB_2_ knockout animals (Figure 6(c)). Collectively, our data show that the absence of CB_2_ leads to rapid and enhanced neutrophil infiltration to the spleen during endotoxemia.

To understand the mechanism, we screened for differentially expressed genes between pooled RNA samples from spleens of wild-type and CB_2_ knockout mice administered with 1 mg/kg LPS for 2 hours using a murine chemokine and chemokine receptors gene array. As shown in Table 2, there were 72 out of 84 genes in the array for which expression was detectable (ΔCt ≤ 12 when normalised to endogenous *Gapdh*) in either wild-type or CB_2_ knockout pooled tissue samples. To identify genes where expression was most altered in the CB_2_ knockout spleens, we applied a cutoff fold change of 2 to the preliminary list (Table 2).

This filter removed 67 genes, and the 5 genes that satisfied the exclusion criteria were further explored: the chemokines *Ccl2*, *Ccl3*, and *Cxcl10* and the neutrophil chemotaxis and degranulation marker *Cmtm6* and the cytokine *Il6*. We proceeded to validate the expression of the genes with qPCR. Our findings confirmed the *Ccl2*, *Cxcl10*, and *Il6* data from the PCR array as we observed a significant (*p* < 0.05) upregulation (*p* < 0.01 for *Ccl3* mRNA levels) of their expression in CB_2_ knockout spleens (Figures 6(d), 6(e), 6(f), and 6(g)). In contrast, *Cmtm* levels were undetectable (data not shown). In conclusion, CB_2_ knockout spleens express higher mRNA levels of CC, CXC chemokines, and *Il6* which suggests a chemokine-dependent mobilisation of neutrophils to this tissue in CB_2_ knockout mice.

## 4. Discussion

In the present study, we report for the first time that CB_2_ deficiency in mice leads to more neutrophils and fewer monocytes in the bone marrow under steady state. Moreover, we observed a CB_2_-dependent suppression of neutrophil recruitment to the spleen at the 2-hour time point of the low-dose endotoxemia model which coincides with elevated levels of MMP-9 in the serum of the animals.

Endotoxemia is frequently employed to model sepsis in animals [47]. The model used in this study recapitulates the main features of endotoxemia, namely, the overwhelming innate immune response and the rapid but transient systemic upregulation of proinflammatory cytokines and chemokines. However, although it has been shown that LPS is pathologically important in human sepsis, endotoxemia models are subject to limitations, and thus their suitability for preclinical trials should be determined by the tested hypothesis. Thus, the findings from these models should be related to the clinical manifestations of sepsis with caution [48, 49]. We therefore decided to use a low-dose endotoxemia model to study the effects of CB_2_ in the context of acute systemic inflammation. Although a low LPS dosage may not exhibit the severe physiological insult present in high-dose endotoxin and bacterial infection models, its advantage is that it can be used to measure the effects of anti-inflammatory drugs or gene deletion without severely affecting the welfare of experimental animals.

Our data demonstrates that the chosen LPS dose and route of administration result in proinflammatory mediator secretion and leukocyte recruitment in the lungs and the peritoneum. Neutrophils were the main immune cell type infiltrating the lungs and the peritoneal cavity consistent with the CXCL1 expression pattern in the periphery. This is in accordance with previous studies that underlined the importance of this chemokine in mediating host defence to pathogens [40]. Interestingly, monocytes were found to migrate only to the lungs of endotoxemic mice. This finding highlights the significance of immune cell composition and architecture of tissues for leukocyte recruitment as previously shown for neutrophils (reviewed in [50, 51]).

A key finding of this study was the lack of changes in systemic levels of proinflammatory mediators in endotoxemic mice in the absence of CB_2_. This is at odds with the literature as CB_2_ has been previously shown to regulate proinflammatory cytokine and chemokine secretion and adhesion molecule expression [24–26]. In contrast, another study using the same animal model of sepsis showed that CB_2_ deficiency was responsible for the dramatic drop in the levels of the same mediators in plasma and peritoneal fluid [30]. The authors found that tissue injury and bacterial burden were also reduced in CB_2_ knockout mice, suggesting that CB_2_ is a receptor that contributes to the pathology of the disease by prolonging host responses. Explanations for this discrepancy could be the different LPS serotypes and dosages used, the route of LPS administration, and the animal species used.

The discrepancies between these papers and our own study may be the choice of the animal model used. The CLP model has been used widely as it resembles the human pathology more reliably than endotoxemia models [52]. However, it is a model of severe inflammation with IL-6 plasma levels being a significant survival predictor [36]. Furthermore, the role of IL-10 in immunosuppression has been highlighted before and provides an explanation for the irreversibility of septic shock and the high mortality rates observed in mice [53, 54]. In our own low-dose LPS model, IL-6 levels were transient, while IL-10 fell beneath the detection limit. Therefore, our results interrogate CB_2_ functions in different pathophysiological conditions from those seen in the CLP model.

The levels of MMP-9 were significantly elevated in the serum of CB_2_ knockout mice. MMP-9 plays an important role in neutrophil transmigration via its role in extracellular matrix degradation and is secreted upon stimulation by chemotactic factors [32, 55]. CB_2_ has been previously shown to affect MMP-9 effector functions in relation to other immune cells. For instance, MMP-9-dependent dendritic cell migration is inhibited upon treatment with the CB_2_-selective agonist Gp1a [56], whereas CB_2_ deficiency leads to increased MMP-9 secretion by macrophages in low-density lipoprotein receptor knockout mice [57]. We report for the first time that CB_2_ regulates MMP-9 levels in a sepsis model, and further investigation is required to determine whether this accounts for differences in neutrophil mobilisation.

One of the main objectives of this study was to assess leukocyte recruitment to peripheral tissues during acute systemic inflammation. Neutrophils reside marginated in tissues, such as the lungs, spleen, and liver where they are in a direct exchange with the circulation [42, 43]. Our results are in seeming disagreement with the work of Tschöp et al. who reported augmented recruitment of neutrophils to the lungs of CB_2_ knockout mice [24]. One possible reason for these differences could be the fact that we have utilised two different models of sepsis which have varying degrees of inflammatory stimulation.

During sepsis, emergency myelopoiesis is triggered in the bone marrow in response to signals from G-CSF released in the blood by the injured endothelium [41, 58, 59]. Hematopoietic stem cells proliferate giving rise to neutrophils that egress from the bone marrow and enter the circulation [41, 60]. Our data rule out a role of CB_2_ in regulating G-CSF-dependent neutrophil egress from the bone marrow; however, another plausible explanation is that CB_2_ controls neutrophil trafficking via direct effects on these cells. In the literature, there are conflicting reports relating to the use of CB_2_ agonists on neutrophil recruitment. For example, the endocannabinoid 2-arachidonoylglycerol is a neutrophil chemoattractant *in vitro* [61]. However, natural and synthetic CB_2_-selective agonists ameliorate neutrophil recruitment in models of inflammation either directly [62–64] or indirectly via the regulation of endothelial proinflammatory gene expression [65].

In our experiments, CB_2_ was shown to control neutrophil recruitment and or retention to the spleen. The upregulation of *Ccl3* and *Cxcl10* in the spleens of CB_2_ knockout mice suggests a chemokine-dependent regulation of neutrophil migration to this organ. CCL3 engages the CCR1 receptor and has been reported to induce calcium alterations in polymorphonuclear leukocytes, while genetic deletion of CCR1 in mice results in a loss of neutrophil mobilisation to CCL3 *in vivo* and impaired killing of *A. fumigatus* conidia [66]. CXCL10, on the other hand, reduces survival in sepsis and contributes to the pathology of sepsis [67]. Both CXCL10 and its cognate receptor CXCR3 are expressed by activated neutrophils and are responsible for their recruitment to the lungs in acute respiratory distress syndrome models [68, 69]. Our data do not exclude the possibility that CB_2_, apart from these chemokines, may also regulate the expression of their respective receptors on neutrophils. However, further studies are needed to determine which pathway and cellular key players are crucial for this effect.

The spleen is an important organ for pathogen clearance by phagocytosis during infection. Phagocytes, such as monocytes/resident macrophages and neutrophils are key players in this process as highlighted by the defects in bacterial and fungal killing observed in splenectomised animals and human patients [45, 70]. Furthermore, splenic neutrophils have been shown to migrate from the marginal zone to the T cell rich area in a CXC chemokine-dependent manner and indirectly induce T cell activation via antigen transfer to dendritic cells [46]. Therefore, we speculate from the results presented in this study that the absence of the CB_2_ receptor may impact the process of LPS clearance.

One possible limitation of our study is that our data were not confirmed with pharmacological inhibition in wild-type littermate animals. To date, three CB2R-selective antagonists have been developed and used extensively *in vitro* and *in vivo*: SR144528, AM630, and JTE907 [15]. These compounds inhibit CB_2_ ligand-induced signalling and displace CB_2_ agonist from CB_2_ in competitive binding assays [71–73]. However, it has also been reported that these antagonists display nonspecific activation of ion channels and CB1R [72, 74–77] and exhibit inverse cannabimimetic effects when administered by themselves *in vivo* [71, 72]. For this reason, we decided to restrict our study to a biological comparison between WT and CB2R^−/−^ mice in endotoxemia.

**Figure 7:**
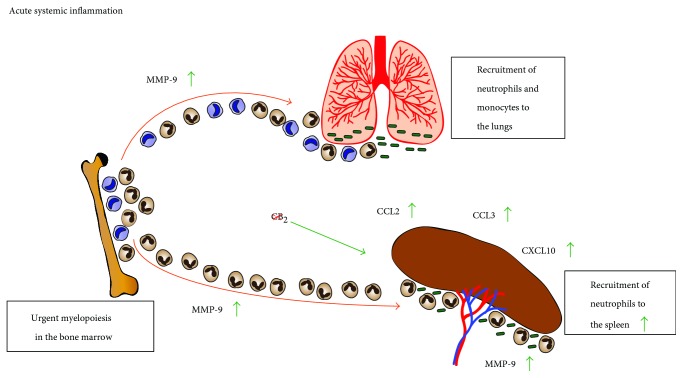
Proposed model for CB_2_ function in endotoxemia. During acute systemic inflammation CB_2_ suppresses neutrophil recruitment to the spleen. In the absence of this GPCR, the serum levels of the metalloproteinase MMP-9 are elevated and *Ccl2*, *Ccl3*, *Cxcl10*, and *Il6* in the spleen are upregulated. As a result CB_2_ knockout, neutrophils follow the chemokine gradient and migrate to the spleen in higher numbers than in control littermate animals.

In summary, we found that the lack of this GPCR leads to enhanced retention of neutrophils and increased release of monocytes in the bone marrow under steady state. We highlight a critical role for CB_2_ in regulating neutrophil infiltration to the spleen during acute systemic inflammation (Figure 7). A potential mechanism for this effect is the increased secretion of MMP-9 and *Ccl3*/*Cxcl10* expression in the spleens of CB_2_ knockout mice. Taken together, we propose a novel role for CB_2_ in suppressing neutrophil migration to lymphoid organs under inflammatory conditions which we believe warrants further investigation.

## Figures and Tables

**Table 1:** Primers used for detection of proinflammatory mediator expression in murine lungs.

Gene	Primer	Primer sequence (5′ → 3′)
*Mm_Ccl2*	Sense	CAGCACCTTTGAATGTGAAGTTG
	Antisense	TGCTTGAGGTGGTTGTGGAA
*Mm_Cxcl1*	Sense	AAGTCATAGCCACACTCAAG
	Antisense	CAGACAGGTGCCATCAGA
*Mm_Actg1*	Sense	CCAACAGCAGACTTCCAGGATT
	Antisense	CTGGCAAGAAGGAGTGGTAACTG

**Table 2:** Chemokine and receptor mRNA expression in spleens of wild-type and CB_2_ knockout mice. Male C57BL/6J mice (8–10 weeks old) were administered with 1 mg/kg LPS for 2 hours and were sacrificed to harvest spleens. The tissues were homogenised, RNA was extracted and reverse transcribed to cDNA. Pooled cDNA from 9 wild-type or CB_2_ knockout mice was used in murine chemokine and receptor PCR arrays. Data are normalised to endogenous *Gapdh* levels and presented as 2^(−ΔCt).

	WT	KO	Fold change (KO/WT)
*c5ar1*	0.026	0.03	0.141
*ccl11*	0.06	0.061	0.022
*ccl12*	0.029	0.061	1.083
*ccl17*	0.059	0.061	0.032
*ccl19*	3.788	3.914	0.033
*ccl2*	0.247	1.01	3.087
*ccl20*	0.028	N/A	−1
*ccl22*	0.028	0.03	0.072
*ccl25*	0.029	0.03	0.03
*ccl26*	N/A	0.026	N/A
*ccl3*	0.059	0.245	3.159
*ccl4*	0.471	0.967	1.052
*ccl5*	0.471	0.969	1.058
*ccl6*	0.03	0.062	1.062
*ccl7*	0.119	0.246	1.058
*ccr1*	0.029	0.029	0
*ccr10*	0.029	N/A	−1
*ccr2*	0.027	0.031	0.151
*ccr3*	0.026	0.029	0.098
*ccr4*	0.028	0.03	0.042
*ccr5*	0.061	0.062	0.01
*ccr6*	0.243	0.492	1.023
*ccr7*	0.242	0.245	0.011
*ccr8*	N/A	0.03	N/A
*ccr9*	N/A	0.029	N/A
*ackr4*	N/A	0.028	N/A
*ccrl2*	0.059	0.122	1.066
*cmklr1*	N/A	0.03	N/A
*cmtm3*	0.062	0.064	0.035
*cmtm4*	0.03	N/A	−1
*cmtm6*	0.23	1.01	3.39
*cx3cl1*	0.028	0.028	0.029
*cx3cr1*	0.028	0.027	−0.024
*cxcl1*	0.12	0.123	0.028
*cxcl10*	1.893	7.87	3.158
*cxcl11*	0.027	0.03	0.089
*cxcl12*	0.06	0.062	0.03
*cxcl13*	0.119	0.245	1.056
*cxcl15*	0.029	0.029	−0.006
*cxcl16*	0.03	0.031	0.033
*cxcl2*	0.121	0.246	1.028
*cxcl3*	0.028	0.03	0.087
*cxcl5*	0.987	2.035	1.062
*cxcl9*	0.029	0.063	1.138
*cxcr1*	N/A	0.028	N/A
*cxcr2*	N/A	0.03	N/A
*cxcr3*	0.028	0.028	0.014
*cxcr4*	0.059	0.031	−0.482
*cxcr5*	0.119	0.246	1.059
*cxcr6*	0.029	0.061	1.116
*ackr3*	N/A	0.029	N/A
*ackr1*	0.028	0.031	0.12
*fpr1*	N/A	0.031	N/A
*hif1a*	0.06	0.123	1.064
*ifng*	0.031	0.032	0.026
*il16*	0.059	0.061	0.025
*il1b*	0.119	0.244	1.048
*il4*	N/A	0.029	N/A
*il6*	0.057	0.244	3.284
*itgam*	0.028	0.031	0.108
*itgb2*	0.12	0.247	1.067
*mapk1*	0.059	0.122	1.069
*mapk14*	0.061	0.124	1.051
*pf4*	0.058	0.06	0.034
*ppbp*	0.12	0.124	0.034
*slit2*	N/A	0.026	N/A
*tgfb1*	0.119	0.245	1.061
*tlr2*	0.03	0.031	0.041
*tlr4*	0.028	0.029	0.034
*tnf*	0.03	0.061	1.048
*xcl1*	0.029	0.03	0.036
*xcr1*	0.028	N/A	−1

## Abbreviations

CB_1_: Cannabinoid receptor 1
CB_2_: Cannabinoid receptor 2
CLP: Caecal ligation and puncture
Ct: Cycle threshold
G-CSF: Granulocyte colony stimulating factor
GPCR: G protein-coupled receptor
i.p.: Intraperitoneally
PFA: Paraformaldehyde.
